# The management of pulmonary hypertension in children

**DOI:** 10.1136/adc.2007.120493

**Published:** 2008-04-01

**Authors:** S G Haworth

## Abstract

Pulmonary hypertension is relatively common in children and has many causes. The management of the condition has changed dramatically in the past 5 years with the introduction of new medicines. However, diagnosis, investigation and choice of therapy remain a challenge. In 2002 the United Kingdom Pulmonary Hypertension Service for Children was established and this has become the mainstay of management in this country. This service, based at Great Ormond Street Hospital for Children, provides advice, expertise and infrastructure support for the most severely affected patients, particularly those with idiopathic pulmonary arterial hypertension for whom chronic intravenous prostacyclin remains the most effective medication. New medicines are being developed which, rather than focussing on dilating a diseased pulmonary vascular bed, aim to structurally remodel the pulmonary vasculature towards normal.

Pulmonary hypertension is more common in children than in adults. A modest elevation in pulmonary arterial pressure (PAP) is tolerable, but a high pressure is associated with obstructive pulmonary vascular disease leading to right heart failure and death. Our perspective on this condition has changed dramatically since I wrote an editorial for this journal in 1998.^1^ Improved diagnostic techniques facilitate assessment, drug discoveries have revolutionised management and the establishment of a UK Service for the Care of Children with Pulmonary Hypertension has facilitated diagnosis and management and provided infrastructure support to improve the quality of life of the most severely affected children. We now have a clinically useful classification of pulmonary hypertension which includes all the types of pulmonary hypertension encountered in childhood (box 1).^2^ We have also established internationally recognised diagnostic and management guidelines.^3^ ^4^ This review is based on the experience gained in establishing and running the UK Service for Children, a service commissioned by the Specialist Commissioning Group. The service is a structured clinical network designed to treat children with pulmonary hypertension rapidly and to provide immediate access to new medicines.

**Box 1 Classification of pulmonary hypertension**

1. Pulmonary arterial hypertension (PAH)

- 1.1 Idiopathic (IPAH)
- 1.2 Familial (FIPAH)
- 1.3 Associated with (APAH)
-
  - 1.3.1 Connective tissue disease
  - 1.3.2 Congenital systemic to pulmonary shunts
  - 1.3.3 Portal hypertension
  - 1.3.4 HIV
  - 1.3.5 *Drugs and toxins*
  - 1.3.6 Other
- 1.4 Associated with significant venous or capillary involvement
-
  - 1.4.1 Pulmonary veno-occlusive disease
  - 1.4.2 Pulmonary capillary haemangiomatosis
- 1.5 Persistent pulmonary hypertension of the newborn

2. Pulmonary hypertension associated with left heart diseases

- 2.1 Left sided atrial or ventricular disease
- 2.2 Left sided valvular heart disease

3. Pulmonary hypertension associated with respiratory disease and/or hypoxia

- 3.1 Chronic obstructive pulmonary disease
- 3.2 Interstitial lung disease
- 3.3 Sleep disordered breathing
- 3.4 Alveolar hypoventilation disorders
- 3.5 *High altitude*
- 3.6 Developmental abnormalities

4. *Pulmonary hypertension due to chronic thrombotic/embolic disease*

5. Miscellaneous

- Tumour, and others

Indicated in *italics* are the only conditions NOT to have been encountered in the UK Pulmonary Hypertension Service for Children.

Pulmonary hypertension is defined as a mean PAP equal to or greater than 25 mm Hg at rest or 30 mm Hg on exercise, a definition which applies to all but the youngest infants. The aetiology is more diverse in paediatric than adult patients (box 1). Pulmonary arterial hypertension is much more common than venous hypertension, particularly in childhood, and the new therapies target arterial rather than venous disease. Pulmonary arterial hypertension is subdivided into idiopathic pulmonary arterial hypertension (IPAH), formerly known as primary pulmonary hypertension, and associated pulmonary arterial hypertension (APAH) when associated with other disorders such as congenital heart disease, connective tissue or lung disease.^2^ There is as yet no evidence of ethnic variability in the different types of pulmonary hypertension seen in British children.

In 1998 the only drugs available to treat pulmonary hypertension were calcium channel antagonists and intravenous epoprostenol, although epoprostenol was only shown to be efficacious in children with IPAH in 1999.^5^ Now there are effective endothelial receptor antagonists, phosphodiesterase inhibitors and prostacyclin analogues, the first two groups of drugs being effective orally.

## DIAGNOSTIC STRATEGY

### Clinical suspicion of pulmonary hypertension (fig 1)

Pulmonary hypertension should be suspected in any child who is unduly short of breath, tires easily or is syncopal when there is no evidence of heart or lung disease. Children occasionally complain of chest pain. The disorder should also be suspected in those known to suffer from these diseases when increasing shortness of breath cannot be explained by the underlying disease process itself. The physical signs of pulmonary hypertension include a left parasternal, right ventricular lift, an accentuated pulmonary component of the second heart sound and sometimes cool extremities. A diastolic murmur of pulmonary regurgitation may be present, but signs of overt right heart failure are a late event in young children.

### Confirming the clinical suspicion of pulmonary hypertension

**Figure 1:**
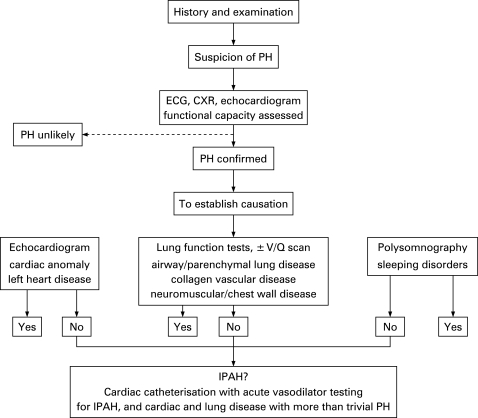
Investigating pulmonary hypertension: diagnostic strategy. CXR, chest *x* ray; PH, pulmonary hypertension; V/Q, ventilation/perfusion.

An accurate and complete diagnosis is essential, remembering that dual pathology is not uncommon in children with pulmonary hypertension. A chest *x* ray, ECG and a transthoracic Doppler echocardiogram are mandatory (fig 1). The classical findings include a chest *x* ray showing enlarged central pulmonary arteries and diminished peripheral pulmonary vascular markings. ECG findings include evidence of right ventricular dilatation and hypertrophy which can be confirmed by transthoracic echocardiography. Doppler interrogation can estimate pulmonary arterial systolic and diastolic pressures. The right ventricular systolic pressure (RVSP) is estimated from the systolic regurgitant tricuspid flow velocity *v* and the estimated right atrial pressure (RAP) in the Bernouille equation: RVSP = 4*v*^2^+RAP. A tricuspid regurgitant jet is present in the majority of pulmonary hypertensive patients. Echocardiographic measures of right ventricular function include the Tei index which is the sum of the isovolumetric contraction time and isovolumetric relaxation time divided by the ejection time. It assesses both systolic and diastolic function. The tricuspid annular plane systolic excursion (TAPSE) correlates with the right ventricular ejection fraction.

Blood tests may show hyperuricaemia, particularly in Eisenmenger syndrome and elevation of brain natriuretic peptide, although less reliably than in adults.^6^

Functional capacity is graded according to the NYHA/WHO classification, from the asymptomatic patient in class I to the severely disabled in class IV. Shortness of breath is a common complaint and an objective assessment of exercise capacity is helpful. In a co-operative child aged 6 years or more the results of a 6 min walk test can be compared with those in normal children of the same age and sex.^7^ Cardiopulmonary exercise testing to determine maximum VO_2_, work rate and anaerobic threshold can also be helpful, but this test should be carried out after the severity of the pulmonary hypertension has been ascertained at cardiac catheterisation, and stressing the child in this way is thought to be safe.

### Classification of pulmonary hypertension: determining causation

If the chest *x* ray excludes parenchymal lung disease, and echocardiography excludes an intracardiac anomaly, then the child probably has IPAH (fig 1). Rarely, pulmonary veno-occlusive disease (PVOD) can masquerade as IPAH. Evaluation must exclude a potentially remedial cause of pulmonary hypertension. The age of the child will obviously determine the ease and success with which certain investigations, such as lung function tests, can be carried out.

Any child suspected of having IPAH or PVOD/pulmonary capillary haemangiomatosis (PCH) should be referred immediately to the UK Pulmonary Hypertension Service for Children.

#### IPAH

##### Presentation

Patients can present throughout childhood, even in infancy. Symptoms vary, are age related and often non-specific. Syncope is relatively common. The commonest misdiagnosis is asthma. Parents report shortness of breath without wheeze, unresponsive to bronchodilator therapy. The familial form of IPAH, which accounts for 6% of all cases, shows genetic anticipation, presentation occurring at a younger age in successive generations.^8^ ^9^ Mutations in the bone morphogenetic receptor-II gene account for the majority of cases of familial IPAH and 26% of sporadic cases.^10^ Pulmonary hypertension also occurs in families with hereditary haemorrhagic telangectasia (HHT) caused by mutations in the activin-like kinase 1 (ALK-1) and endoglin genes.^11^ Taking a careful family history and examining specifically for evidence of HHT is important.

##### Investigation

The systemic arterial oxygen saturation is normal in the absence of an atrial septal defect. Desaturation can occur in the presence of an atrial communication when the PAP, right ventricular and atrial pressures increase and right to left shunting occurs. The ECG findings are characteristic, and include right axis deviation, evidence of right ventricular hypertrophy with or without a strain pattern and right atrial enlargement. In addition to an abnormal chest *x* ray, contrast enhanced spiral CT of the lungs is indicated in older patients to exclude thrombus in the central pulmonary arteries and chronic thrombo-embolic disease. It is also helpful in distinguishing IPAH from PVOD. Ventilation/perfusion scans can be normal in IPAH but may show small peripheral non-segmental perfusion defects. Magnetic resonance imaging can help assess right ventricular function but is not yet part of the routine investigation in children with IPAH. The echocardiogram generally reveals dilated right heart chambers, right ventricular hypertrophy with posterior bowing of the ventricular and, in the absence of an atrial communication, the atrial septum.^12^ The left ventricle may be severely compressed. Right atrial size and left ventricular eccentricity index are predictive of outcome in adults and are routinely assessed in children. Lung function testing can sometimes demonstrate small airways obstruction.

In addition to routine haematology and biochemistry, thyroid function tests, a thrombophilia screen including antiphospholipid antibodies (lupus anticoagulant, anticardiolpin antibodies) and an auto-immune screen are carried out. Children may seroconvert when they are older. Some children have an immunoglobulin deficiency. They may have a low level of antithrombin III, protein S or protein C, which may be genetic in origin or result from a consumption coagulopathy.

Screening of other siblings for the familial form of the disease is necessary.

#### APAH

Successful treatment of the pulmonary hypertension depends on the rapidity with which the physician treating the underlying disease recognises that pulmonary hypertension is a major complicating factor in the clinical picture.

Presentation and the investigation of children with APAH are determined by the underlying disease process. The principle aetiologies are as follows.

##### Chronic hypoxia

Chronic hypoxia leads to pulmonary hypertension. Chronic lung disease of prematurity is a relatively common cause of moderate pulmonary hypertension. Interstitial lung disease, as strictly defined, is uncommon in children, as are the pulmonary complications of connective tissue disease. When pulmonary hypertension does occur in association with connective tissue disease in children, it is usually severe. Of the developmental abnormalities, the commonest association is congenital diaphragmatic hernia. In these patients pulmonary hypertension is relatively common in the neonatal period. It usually abates but can persist indefinitely, be severe and require long term treatment. The extent to which lung disease is causing or contributing to an elevated PAP is revealed by pulmonary function tests, the blood gases, ventilation/perfusion and high resolution CT scans.

##### Persistent pulmonary hypertension of the newborn (PPHN)

This condition is usually self-limiting unless there is a significant irreversible underlying problem such as chronic lung disease of prematurity. Alveolar hypoplasia, usually accompanied by a degree of dysplasia, is signalled by persistent failure to wean off the ventilator and an open lung biopsy may be needed to guide management. PPHN may also be the first indication of IPAH.

##### Congenital heart disease

Sustained pulmonary hypertension is sometimes recognised following a technically successful intra-cardiac repair and can be severe. Typically, the child improves clinically after the repair but then becomes short of breath and tires easily. The more severely affected children are investigated and treated as though they had IPAH. Classical Eisenmenger syndrome in the patient who initially had a large left to right shunt and then slowly developed pulmonary vascular disease with shunt reversal, central cyanosis and clubbing is not usually a problem in childhood. Most patients begin to deteriorate in their teenage years. Any child who appears to have Eisenmenger syndrome should be evaluated carefully to ensure that the cardiac diagnosis is correct and complete and that remedial surgery is not feasible. Sleep studies may be indicated in those with trisomy 21. Paediatric cardiology units are familiar with the small group of children who had a high pulmonary vascular resistance when first seen and were therefore deemed inoperable. In such children the resistance probably remained high from birth. They frequently become symptomatic in childhood and may benefit from medical therapy. Complex surgically naive and post-operative cases for whom curative or palliative surgery is not feasible frequently need reappraisal including cardiac catheterisation with a view to palliation by medical therapy.

##### Liver disease

Pulmonary hypertension is a recognised complication of chronic liver disease. Portal hypertension rather than hepatocellular disease appears to be the main determinant. It is uncommon in childhood but when present can influence the timing of liver transplantation. In adults liver transplantation is contra-indicated if the mean PAP exceeds 35 mm Hg and the pulmonary vascular resistance is greater than 250 dynes/s/cm.^13^

#### PVOD and PCH

These conditions are uncommon. They can present like IPAH, but recognition is important because their management differs from that of IPAH. PVOD usually presents with shortness of breath, sometimes accompanied by small, frequent haemoptysis. The patient may be desaturated, clubbed and have minimal rales on auscultation. The ECG and echocardiogram can be indistinguishable from IPAH but the lung diffusing capacity is lower and high resolution CT scans can be diagnostic to the experienced radiologist. In addition to the expected features of pulmonary hypertension, contrast CT with vascular imaging shows a patchy centrilobular pattern of ground glass opacities, the lobules having a “pavement appearance” because their margins are demarcated by thickened septal lines caused by oedema. Typically, mediastinal lymphadenopathy is prominent. The contrast scan also confirms the echocardiographic findings of unobstructed venous return in the large pulmonary veins. These children are usually very ill indeed and should be listed for transplantation without delay. Cardiac catheterisation may be indicated to confirm the diagnosis. If the child is relatively well and stable, it can be helpful to do an open lung biopsy in order to distinguish those with PCH who may be amenable to treatment for a short period of time, although such treatment with anti-cancer drugs must be regarded as experimental. Medical treatment of PVOD with pulmonary hypertension specific therapies can be hazardous and is contraindicated.

### Cardiac catheterisation to confirm the diagnosis, assess disease severity and guide therapeutic management and the role of lung biopsy

The purpose of cardiac catheterisation in children with pulmonary hypertension is to confirm the diagnosis and to ensure that the conclusions drawn from the non-invasive tests were complete and accurate. The catheter also determines disease severity by determining the PAP accurately, is the only reliable means to date of determining pulmonary vascular resistance and tests the vasoreactivity of the pulmonary vasculature. The main determinant of treatment is the response to vasodilator testing with nitric oxide at cardiac catheterisation. Pulmonary vascular resistance can only be determined using the Fick principle if the pulmonary blood flow can be determined accurately by measuring, rather than assuming, the oxygen consumption.

The risk of both cardiac catheterisation and general anaesthesia are increased in the presence of pulmonary hypertension and therefore the procedure is carefully planned after discussion with the child’s parents. Adequate sedation, optimal ventilation and meticulous attention to acid base status and blood loss is mandatory. The clinical history and the haemodynamic findings may indicate the need for further interventions such as the creation of an atrial septostomy and/or insertion of a Hickman line for continuous infusion of epoprostenol. These procedures are best done under the one anaesthetic, but the family need to be fully informed of their likelihood and significance and consent to these procedures, or not, before the catheterisation study can take place.

Lung biopsy is rarely justified in pulmonary hypertension. The exceptions are suspicion of PVOD or PCH, of alveolar hypoplasia/dysplasia in PPHN, and very rarely in children with complex congenital heart disease in whom it might still be possible to operate.

### Treatment of pulmonary arterial hypertension

Immunisation schedules should be maintained, and young children need respiratory syncytial virus prophylaxis with palivizumab. Anaesthesia for any general surgical or dental procedure requires particular care.

The aim of medical treatment is to dilate the pulmonary vasculature and reverse the abnormal remodelling characteristic of pulmonary vascular disease. The practical difficulties encountered in treating children influence management and include their age, level of understanding, size and in some, the presence of other anomalies. Three signalling pathways are targeted: the prostacyclin, endothelin and nitric oxide pathways.

#### Prostacyclin and its analogues

The most effective therapy is a continuous intravenous infusion of epoprostenol, the sodium salt of prostacyclin. It has a short half-life of 3–5 min, is unstable and the infusion has to be prepared every 24 h. The principle side effects are jaw pain and diarrhoea. The child is dosed according to response. Children need much higher doses than adults. A more stable analogue of prostacyclin, treprostinil, can also be given intravenously but is associated with more prominent side effects, headaches and leg pain. Meticulous care of the Hickman line is essential to prevent local and systemic infections, the latter being extremely uncommon. Treprostinil can also be given by continuous subcutaneous infusion but is painful, the drug stimulating nerve endings and causing induration and sometimes ulceration of the skin.^14^ It is not used in children. Iloprost is a prostacyclin analogue which can be given by inhalation but small, tired children find it difficult to inhale an effective dose every 2 h. The drug is effective for less than 2 h.

#### Endothelin receptor antagonists

The dual endothelin (ET) receptor antagonist bosentan (Tracleer) was the first oral drug shown to be efficacious in IPAH and has been used extensively in this and other types of pulmonary hypertension since its introduction in 2002.^15^ It is efficacious in children.^16^ Its principle side effect is elevation of liver enzymes which necessitates a monthly blood test. Drug interactions can occur. Bosentan decreases effective exposure to warfarin because of induction of CYP3A4 and/or CYP2C9. The newer selective ET-A receptor antagonists, sitaxentan and ambrisentan have not yet been studied in children. Both drugs affect the liver less than bosentan and drug interaction is probably less likely with ambrisentan.

#### Phosphodiesterase inhibitors

Sildenafil was the first drug of this class and is still the most commonly used, particularly in young children with APAH. The principle side effects include erections and systemic hypotension when high doses are used. The dose is 0.5–1 mg/kg/dose, given three to four times a day, rarely more.

#### Anticoagulation

Patients with pulmonary vascular disease are prone to develop thrombosis in situ. Older children are given warfarin and younger ones usually receive aspirin. The INR must be monitored particularly closely in those on endothelin receptor antagonists.

#### Oxygen

Oxygen is a potent pulmonary vasodilator. Nocturnal supplemental oxygen is indicated if there is nocturnal systemic arterial oxygen desaturation and can benefit those with a high PAP.

#### Atrial septostomy

This procedure is indicated in children with IPAH and post-operative pulmonary hypertension suffering from syncope and/or severe right heart failure.^17^ It reduces the effect of a sudden increase in pulmonary arterial and right heart pressures and maintains a left ventricular output.

#### Lung and heart–lung transplantation

The policy of the UK Pulmonary Hypertension Service is to refer children on intravenous epoprostenol for assessment by the transplantation service when they are established on treatment and are still well. They are then reviewed by the transplantation service as indicated.

## WHICH CHILDREN WILL BENEFIT FROM WHICH DRUG?

It is impossible to generalise about who should and should not be treated. All children with IPAH need urgent treatment, but in those with APAH it is the severity of the pulmonary hypertension and the extent to which even a modest increase in pressure influences their well-being and prognosis which determines the need to treat.

### Treating IPAH

Without treatment the expected survival is less than 1 year.^5^ The main determinant of treatment is the response to vasodilator testing with nitric oxide at cardiac catheterisation. In those with a positive response the PAP and PVR (pulmonary vascular resistance) must fall to a near normal level with no fall in cardiac output. Only patients with a positive vasodilator response can be treated with a calcium channel antagonist. This applies to less than 10% of those with IPAH. Children who improve and are stable on a calcium channel antagonist need repeat cardiac catheterisation after 1 year or less, because they can become resistant to the drug at any time and need escalation of therapy before they deteriorate. The majority of negative responders present in NYHA/WHO class III–IV and frequently need to be started on intravenous epoprostenol therapy immediately. In a minority it is feasible to initiate therapy with an endothelial receptor antagonist. Most children however, require dual therapy. Sildenafil has not been proven to be efficacious in children. Syncopal children need an urgent atrial septostomy. After discharge from hospital close monitoring, principally by echocardiography, is mandatory since urgent intensification of therapy is frequently necessary.

### Treating APAH

#### Congenital heart disease

Children with severe post-operative pulmonary hypertension are treated like those with IPAH. Symptomatic children with classical Eisenmenger syndrome are treated either with an endothelin receptor antagonist or sildenafil, according to age, sex and maturation. Children with a high PVR who have never been operable are usually more symptomatic at an early age and need similar treatment.

#### Chronic lung and connective tissue disease

These patients are treated with bosentan which is thought to have an anti-fibrotic effect in addition to treating pulmonary vascular disease. Young and less severely affected children receive sildenafil. Those with connective tissue disease may require intravenous epoprostenol.

#### PPHN persisting beyond the neonatal period

Children with a modest increase in PAP are treated with sildenafil unless it is apparent that resolution is not occurring when therapy is intensified.

In both chronic lung disease and PPHN the aim is to encourage normalisation of the pulmonary vasculature and so be able to stop treatment with relatively new, powerful drugs whose long term effects on children are unknown.

#### Bone marrow transplantation

Pulmonary hypertension can occur as a result of the condition indicating bone marrow transplantation, be a complication of transplantation or be an incidental finding. In any event, treatment with bosentan is contraindicated because it interacts with cyclosporine and tacrolimus. Sildenafil can be given to the less severely affected, but intravenous epoprostenol may be indicated.

### Quality of life

All children are encouraged to return to school quickly. For those on intravenous therapy, dedicated school carers are given appropriate training by the clinical nurse specialists of the UK Pulmonary Hypertension Service who visit the school regularly. All drugs and equipment for intravenous, inhalational and oral therapy are delivered directly to the child’s home. Psychological support may be necessary, particularly with respect to managing relationships in a family with a chronically sick child. The patients’ organisation, The Pulmonary Hypertension Association UK, is a great source of comfort and support. Hospice care also helps many families. It is the responsibility of the UK Pulmonary Hypertension Service for Children to establish support networks in the community for these families.

Improving quality of life must be the first priority when treating an incurable disease. In the Quality of Life questionnaires completed by the parents and older children cared for by the UK Pulmonary Hypertension Service the scores for physical performance were low, as expected.^18^ The psychosocial scores were significantly higher, almost normal, a result which probably indicates the extent to which expectations change in chronic disease.

### Lung transplantation

Patients who fail to respond to medical therapy are offered the possibility of lung transplantation. The predicted survival for lung transplantation in children is 4.3 years with a 75% survival at 1 year.^19^

### Results of treatment

In IPAH, the UK Pulmonary Hypertension Service showed survival figures of 84% at 1 year and 76% at 3 years.^18^ In APAH the survival rates were 89% at 1 year and 79% at 3 years. These figures compare favourably with those in international adult and paediatric studies.

### New and emerging therapies

The therapeutic goal is the remodelling of the pulmonary vasculature back to normal, the restoration of endothelial function and the growth of new peripheral pulmonary arteries. New modes of administration, new formulations and new analogues of existing drugs, new endothelin receptor antagonists and new PDE 1/5/6 plus PDE-3 and PDE-1 inhibitors are in development now. Statins, RhoA inhibitors, anti-growth factor drugs, metalloproteinase inhibitors, K channel openers, stem cell and gene therapy and vasoactive intestinal peptide are all under investigation. At present we can stabilise patients for many years, but new, radical medicines used in combination offer the hope of cure and greater promise of tailoring the treatment to the individual child.
